# H_2_O_2_ Activation and Alkane Oxidation by Copper Complexes With R‐dpa N_3_‐Tridentate Ligands: The Complex‐Based Dicopper Active Species as a Key Feature in the Efficient Alkane Oxidation

**DOI:** 10.1002/chem.70985

**Published:** 2026-04-16

**Authors:** Kyosuke Fujikawa, Momoe Kawahashi, Alexander Granichny, Siegfried Schindler, Hiroaki Kitagishi, Masahito Kodera

**Affiliations:** ^1^ Department of Molecular Chemistry and Biochemistry Doshisha University Kyotanabe Kyoto Japan; ^2^ Institute of Inorganic and Analytical Chemistry Justus Liebig University Gessen Giessen Germany

## Abstract

Three Cu(II) complexes [Cu(Rdpa)(MeCN)](ClO_4_)_2_ (**1^R^**, R = Me, Phe, *t*‐Bu) were prepared with N_3_‐tridentate ligands, based on bis(2‐pycolyl)alkylamine (R‐dpa), to examine the mechanism of cyclohexane (CyH) oxidation with H_2_O_2_ catalyzed by **1^R^**. The crystal structures of **1^R^** revealed that the steric hindrance of the R group strongly affects the bridging structure. Reaction analysis using the HO• trapping reagent DMPO revealed that the CyH oxidation proceeds in two reaction pathways via the formation of HO• and complex‐based active species. The H_2_O_2_ activation of **1^R^** was monitored by low‐temperature stopped‐flow techniques, where hydroperoxo Cu(II) and bis‐μ‐oxo Cu(III)_2_ species were formed as key intermediates, depending on the ease of bridging structure formation. Based on these results, two H_2_O_2_ activation pathways (Paths 1 and 2) are proposed. In Path 1, the hydroperoxo Cu(II) intermediate forms HO• as the active species, which shortens the catalyst lifetime due to nonselective oxidation of the supporting ligand. In Path 2, the bis‐μ‐oxo Cu(III)_2_ intermediate forms a μ‐oxyl radical (μ–O•) bridged Cu(II)_2_ complex as a relevant complex‐based active species which prolongs the catalyst lifetime to achieve a large turnover number (TON) in CyH oxidation. These findings pave the way for developing efficient Cu complex catalysts for alkane oxidation.

## Graphical abstract

Two reaction pathways, Paths 1 and 2, are involved in H_2_O_2_ activation by Cu complexes with N_3_‐tridentate ligands, depending on the ease of bridging structure formation. In Path 1, hydroxy radical (HO•) is formed from a hydroperoxo Cu(II) intermediate. In Path 2, a relevant complex‐based μ‐O• bridged Cu(II)_2_ active species is formed from a bis‐μ‐oxo Cu(III)_2_ intermediate.

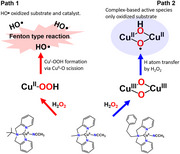

## 1 Introduction

Alkane oxidation catalyzed by Cu complexes is important for the synthetic applications [1, 2, 3, 4]. A chemical understanding of alkane oxidation by Cu‐containing enzymes such as lytic polysaccharide monooxygenases (LPMO) [5, 6, 7] and particulate methane monooxygenases (pMMO) [8, 9, 10, 11, 12, 13, 14] serves to develop Cu catalysts for alkane oxidation. LPMOs promote cellulose degradation with hydrogen peroxide (H_2_O_2_) as an oxidant, where a Cu‐oxyl radical (Cu(II)‐O•) and a hydroxyl radical (HO•) have been proposed as the active species [15]. Although the structure of pMMO has been reported based on single‐crystal x‐ray analysis [16, 17] and cryo‐EM [18, 19] studies, it remains unclear whether the active site is mono‐ or dicopper, and the O_2_‐activation mechanism is still unknown. Yoshizawa reported a DFT study on O_2_‐activation by pMMO, assuming a dicopper active site. In this model, a Cu(I)_2_ site reacts with O_2_ to form a μ‐η^2^:η^2^‐peroxo Cu(II)_2_ complex, which is reduced by one electron through H‐atom transfer from Tyr374 located in the second coordination sphere. This process yields a μ‐oxyl radical (μ‐O•) Cu(II)_2_ complex (Cu(II)_2_‐μ‐OH‐μ‐O•), which acts as the active species for methane oxidation [20]. Thus, Cu(II)‐O• [21] and Cu(II)_2_‐μ‐OH‐μ‐O• have been proposed as active species to cleave a strong C─H bond. Moreover, Solomon and Larcher reported DFT studies on methane oxidation by Cu‐exchanged zeolite, proposing that di‐ and tri‐Cu μ‐O• species serve as the active species [22, 23, 24].

**CHART 1:**
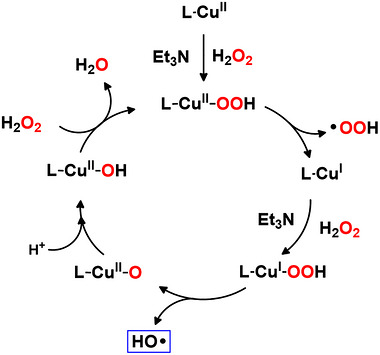
Fenton‐type reaction proposed for H_2_O_2_ activation by Cu(II) complexes of ligands derived from tpa [27].

The substrate oxidations with H_2_O_2_ catalyzed by Cu(II) complexes with N_4_‐tetradentate ligands have been well studied. These Cu complexes demonstrated high catalytic activity in cyclohexane (CyH) oxidation with H_2_O_2_, giving higher product yields than their Fe counterparts [25]. Additionally, a Cu(II) complex with tris(2‐pycolyl)amine (tpa) effectively catalyzed benzene oxidation with H_2_O_2_ [26]. These reactions proceed via Fenton‐type pathways, forming HO• and HO_2_• radicals. Karlin clarified the mechanism using Cu(II) complexes of tpa‐derived ligands (L) [27]. As shown in Chart 1, the Cu complex reacts with H_2_O_2_ in the presence of Et_3_N to form [LCu(II)O_2_H]^+^, followed by energetically favorable Cu(II)─O bond scission, leading to a Cu(I) complex and •O_2_H. The resulting Cu(I) complex reacts with H_2_O_2_ to form [LCu(I)O_2_H], which undergoes energetically favorable O─O bond scission to give [LCu(II)OH]^+^ and HO•. Thus, the H_2_O_2_ activation catalyzed by Cu complexes with N_4_‐tetradentate ligands proceeds in a Fenton‐type reaction to yield HO• and HO_2_• radicals.

**CHART 2:**
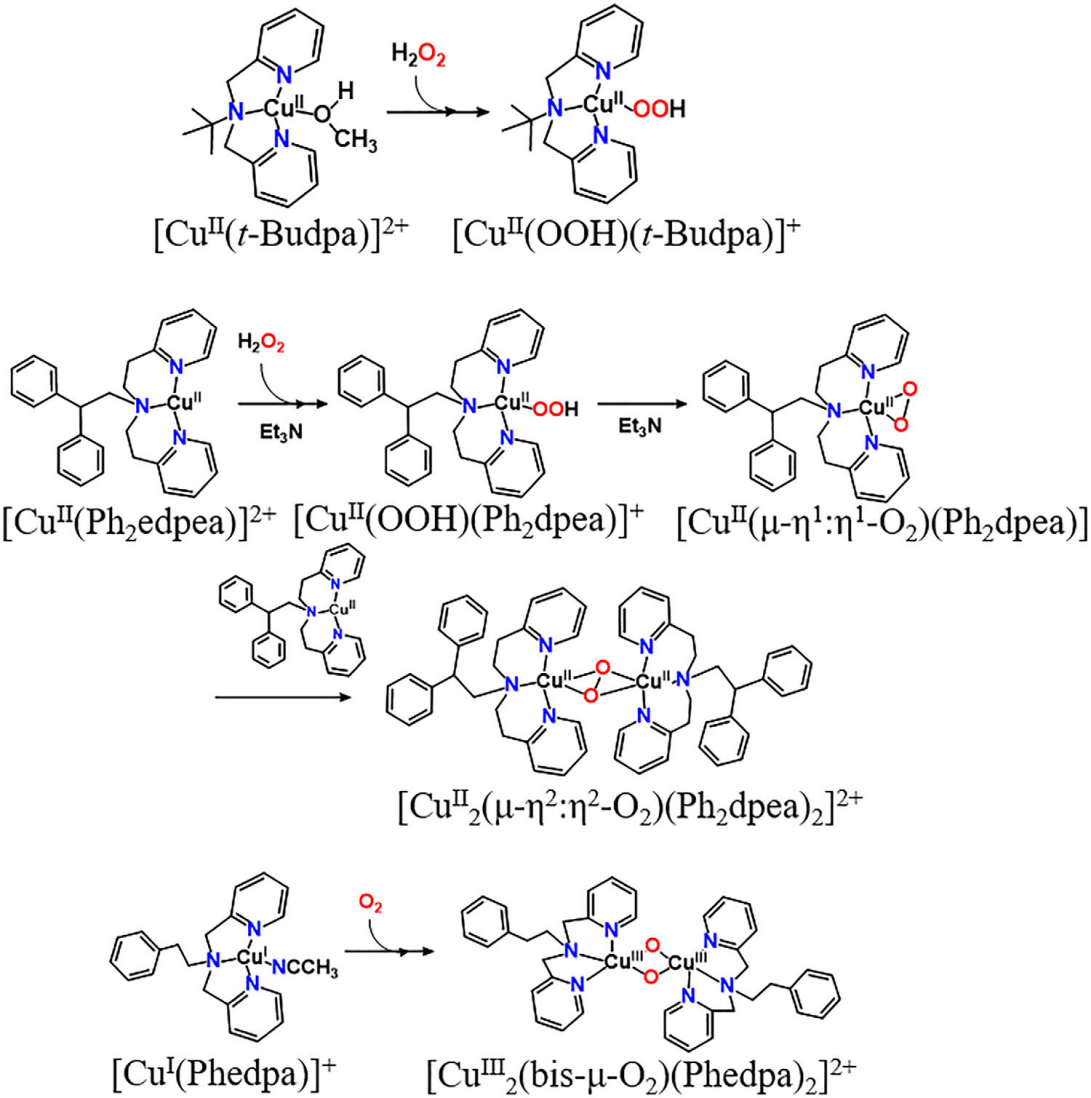
Various intermediates have been reported for the reactions of H_2_O_2_ or O_2_ with the Cu complexes of N_3_‐tridentate ligands, *t*‐Budpa, Phedpa, and Phe_2_dpea [28, 29, 30].

Meanwhile, Cu complexes with N_3_‐tridentate ligands, *N*‐alkyldi(2‐pycolyl)amine (Rdpa, R = *t*‐Bu, Phe) and *N*‐diphenylethyldi(2‐pyridylethyl)amine (Ph_2_edpea) have been shown to react with H_2_O_2_ or O_2_ to form various intermediates spectroscopically identified (Chart 2). Masuda reported that a hydroperoxo Cu(II) complex [Cu(II)(*t*‐Budpa)O_2_H]^+^ was formed upon reaction of [Cu(II)(*t*‐Budpa)]^2+^ with H_2_O_2_ [28]. Itoh showed that a hydroperoxo Cu(II) complex [Cu(II)(Ph_2_edpea)O_2_H]^+^ was formed upon reaction of [Cu(II)(Ph_2_edpea)]^2+^ with H_2_O_2_ and converted to a μ‐η^2^:η^2^‐peroxo Cu(II)_2_ complex [Cu(II)_2_(μ‐η^2^:η^2^‐O_2_)(Ph_2_edpea)_2_]^2+^ via a μ‐η^1^:η^1^‐peroxo Cu(II) complex [Cu(II)(μ‐η^1^:η^1^‐O_2_)(Ph_2_edpea)] [29], and further reported that a Cu(I) Phedpa complex formed a bis‐μ‐oxo Cu(III)_2_ complex [Cu(III)_2_(μ‐O)_2_(Phedpa)_2_]^2+^ upon reaction with O_2_ [30]. These intermediates may be converted to the complex‐based active species, Cu(II)‐O• and Cu(II)_2_‐μ‐OH‐μ‐O•, based on the proposed O_2_ activation mechanism of LPMO and pMMO. However, since the substrate oxidations with H_2_O_2_ catalyzed by the Cu complexes with N_3_‐tridentate ligands have been less investigated, the active species remain unclear [31].

Here, we synthesized [Cu(Rdpa)(MeCN)](ClO_4_)_2_ (**1^R^**) (R = Me, Phe, *t*‐Bu) to examine CyH oxidation with H_2_O_2_ catalyzed by **1^R^**. Reaction analysis revealed that both HO• and complex‐based active species participate. Moreover, x‐ray crystallography and low‐temperature stopped‐flow spectroscopy showed that H_2_O_2_ activation depends on the steric effect of the R group on Rdpa. It was specifically found that promoting the formation of complex‐based active species, while suppressing HO• formation, leads to an increased turnover number (TON) of **1^R^** in the CyH oxidation.

## 2 Experimental Section

### 2.1 Materials

All ordinary reagents were purchased and used as received unless noted. MeCN was dried over P_2_O_5_ and distilled. Et_3_N was purified by distillation from sodium and kept on NaOH. The H_2_O_2_ concentration was determined by titration using KMnO_4_. The Cu(II) complex with tpa (tris(2‐pyridylmethyl)amine), [Cu(MeCN)(tpa)](ClO_4_)_2_ (**2**), tridentate ligands, Rdpa (R = Me, Phe, *t*‐Bu), and the Cu(I) salt, [Cu(MeCN)_4_](ClO_4_), were prepared according to the literature, respectively [28, 32, 33, 34, 35]. 5,5‐dimetyl‐1‐pyrroline‐*N*‐oxide (DMPO) was used as a radical trap reagent [36].

### 2.2 Measurements

Electrospray ionization mass spectra (ESI‐MS) and Cold‐spray ionization mass spectra (CSI‐MS) were recorded on a JEOL JMS‐T100CS spectrometer. ^1^H NMR spectra were recorded on a JEOL ECA‐500RX spectrometer using Me_4_Si. Elemental analyses (C, H and N) were carried out on a Perkin‐Elmer Elemental Analyzer 2400 II. GC analyses were performed on a Shimadzu GC‐2014 gas chromatography equipped with a GL Science InertCap 1701 capillary column (60m 0.25 mm). Low‐temperature stopped‐flow UV–vis spectra were performed by applying a HI‐TECH SF‐61SX2 stopped‐flow unit (TgK Scientific, Bratford‐on‐Avon, UK). The collected data were processed using Kinetic Studio version 5.02 Beta. The procedure for kinetic measurements was described in detail in previous work [37]. Cyclic voltammetry (CV) was measured on an ALS model 703E dual electrochemical analyzer and GAMRY INTERFACE 1000.

### 2.3 Synthesis of [Cu(Medpa)(MeCN)](ClO_4_)_2_ (1^Me^)

To a 0.8 mL MeCN solution of Cu(ClO_4_)_2_ • 6H_2_O (52.0 mg, 0.140 mmol), a 0.4 mL of Medpa (27,2 mg, 0.128 mmol) MeCN solution was added and stirred for 15 min. The reaction mixture was added to 100 mL of Et_2_O to give a blue precipitate. The solid was collected by filtration and dried in vacuo. **1^Me^** was recrystallized from MeCN/Et_2_O (37.4 mg, 0.0724 mmol, yield 57%), which is suitable for x‐ray analysis. The crystals were used for oxidation reaction, spectroscopic, and electrochemical measurements. Elemental analysis of **1^Me^**: Calcd. for C_15_H_18_Cl_2_CuN_4_O_8_: C 34.86, H 3.51, N 10.84; Found: C 35.12, H 3.38, N 10.65. ESI‐MS (MeCN *m/z*, positive mode): Calcd. for [**1^Me^** − 2ClO_4_ + Cl]^+^ 311.03; Found: 310.96.

### 2.4 Synthesis of [Cu(Phedpa)(MeCN)](ClO_4_)_2_ (1^Phe^)

To a 0.6 mL MeCN solution of Cu(ClO_4_)_2_ • 6H_2_O (40.6 mg, 0.110 mmol), a 0.9 mL MeCN solution of Phedpa (28.8 mg, 0.0949) was added and stirred for 30 min. The reaction mixture was concentrated, and precooled Et_2_O (about −40°C) was added to the solution, causing the formation of a blue‐colored precipitate. The solid was collected by filtration and dried in vacuo to give a blue‐colored powder (44.8 mg, 0.0738 mmol, yield 78%). **1^Phe^** was recrystallized from MeCN/Et_2_O, leading to crystals suitable for x‐ray analysis. The crystals were used for oxidation reaction, spectroscopic, and electrochemical measurements. Elemental analysis of **1^Phe^**: Calcd. for C_22_H_24_Cl_2_CuN_4_O_8_: C 43.54, H 3.99, N 9.23; Found: C 43.22, H 3.80, N 9.10. ESI‐MS (MeCN *m/z*, positive mode): Calcd. for [**1^Phe^** − ClO_4_]^+^ 465.05, Found: 465.02.

### 2.5 Synthesis of [Cu(*t*‐Budpa)(MeCN)](ClO_4_)_2_ (1*^t^* ^‐Bu^)

To a 1 mL MeCN solution of Cu(ClO_4_)_2_ • 6H_2_O (34.2 mg, 0.0874 mmol), 1 mL of a *t*‐Budpa (20.3 mg, 0.0794 mmol) MeCN solution was added and stirred for 30 min. The reaction mixture was concentrated to ca. 0.6 mL, and vapor diffusion of Et_2_O into the solution gave a deep blue crystal of **1*^t^* ^‐Bu^** suitable for x‐ray analysis. After drying in vacuo, **1*^t^* ^‐Bu^** (33.4 mg, 0.0579 mmol, yield 73%) was obtained as a blue powder, which was used for oxidation reaction, spectroscopic, and electrochemical measurements. Elemental analysis of [**1*^t^* ^‐Bu^**—MeCN + H_2_O]: Calcd. for C_16_H_23_Cl_2_CuN_3_O_9_: C 35.86, H 4.33, N 7.84; Found: C 35.65, H 4.22, N 7.75. ESI‐MS (MeCN *m/z*, positive mode): Calcd. for [**1*^t^* ^‐Bu^** − MeCN − ClO_4_]^+^: 417.05, Found: 417.04.

### 2.6 Structure Determination of Single Crystals

The crystal structures of **1^R^** were determined with a Rigaku R‐AXIS RAPID diffractometer using multi‐layer mirror monochromated Cu–Kα radiation. The data were collected at a temperature of −170°C ± 1°C to a maximum 2*θ* value of 136.48°. The linear absorption coefficients, *μ*, for Cu─K*α* radiation are 3.968 and 2.782 mm^−1^. An empirical absorption correction was applied. The data were corrected for Lorentz and polarization effects. Crystallographic data reported in this manuscript have been deposited with the Cambridge Crystallographic Data Centre as supplementary publication No. CCDC‐2497336 for **1^Me^**, CCDC‐2497337 for **1^Phe^**, and CCDC‐2497338 for **1*^t^* ^‐Bu^**. Copies of the data can be obtained free of charge via the CCDC Website.

### 2.7 General Procedure for the CyH Oxidation with H_2_O_2_ Catalyzed by **1^R^**

To a solution of cyclohexane (1.684 g (20 mmol)), 40 µL of nitrobenzene as an internal standard in MeCN (22 mL) was added 1 mL of MeCN solution of **1^R^** (2 µmol) or **2** (2 µmol), 50 µL of MeCN solution of Et_3_N (10 µmol), and 1.0 mL of aqueous H_2_O_2_ (10 mmol) with vigorous stirring at 50°C under N_2_. The reaction was continued until the increase in the products was saturated. To determine the products at each reaction time, 0.5 mL of the reaction mixture was taken and treated with 60 mg of triphenyl phosphine (PPh_3_) for 30 min. After filtration of the insoluble solid, the products were analyzed by GC.

### 2.8 Effects of a Radical Trap Reagent DMPO on the CyH oxidation

To examine the effect of the radical trap reagent DMPO, the CyH oxidations catalyzed by **1^R^** were carried out in the presence of DMPO (120 µmol) under the reaction conditions described above. The products were analyzed by GC as described above.

### 2.9 Effect of Slow Addition of H_2_O_2_ on the CyH oxidation

To investigate the effect of the concentration of H_2_O_2_ on the CyH oxidation, H_2_O_2_ was added stepwise (1 mmol × 10 times, every 5 min) to the reaction system. Reaction conditions were set as described above, except for the way of H_2_O_2_ addition. The products were analyzed by GC as described above.

### 2.10 Analysis of the reaction of **1^R^** with H_2_O_2_ by Low‐Temperature Stopped‐Flow UV–Vis Spectroscopy

To investigate the reaction of **1^R^** with H_2_O_2_, complex solutions (1 mM) in anhydrous degassed acetone were prepared in an Ar‐filled glovebox. H_2_O_2_ (5 mM) and Et_3_N (5 mM) solutions in regular acetone were prepared outside the glovebox. These solutions were added to two separate gastight syringes, and both syringes were attached to the stopped flow unit. Measurements were carried out at low temperatures from −82°C to −30°C.

### 2.11 Analysis of the Reaction of Cu(I) Complexes of Rdpa with O_2_ by Low‐Temperature Stopped‐Flow UV–Vis Spectroscopy

To investigate the reaction of Cu(I) complexes of Rdpa with O_2_, [Cu(MeCN)_4_](ClO_4_) (1 mM) in anhydrous, degassed acetone was prepared in an argon‐filled glovebox. Rdpa solutions were prepared in the same way. The Rdpa solution was saturated with pure O_2_ (11.4 mM) [25], and both syringes were attached to the stopped flow unit. Measurements were carried out at −82°C.

### 2.12 Cyclic Voltammetry of **1^R^**

MeCN solutions (10 mL) of **1^R^** (0.1 mM) in the presence of TBAP (0.1 M) were placed in an electrochemical cell. The measurements were carried out at 23°C ± 0.2°C under N_2_. Glassy carbon (0.07 cm^2^), Pt wire, and Ag/AgNO_3_ were used as the working electrode, counter electrode, and reference electrode, respectively. Scan rate was 25 mV/s, and sensitivity was 1 mA/V.

## 3 Results and Discussion

### 3.1 Synthesis and Crystal Structures of Cu(II) Complexes of **1^R^**

**FIGURE 1:**
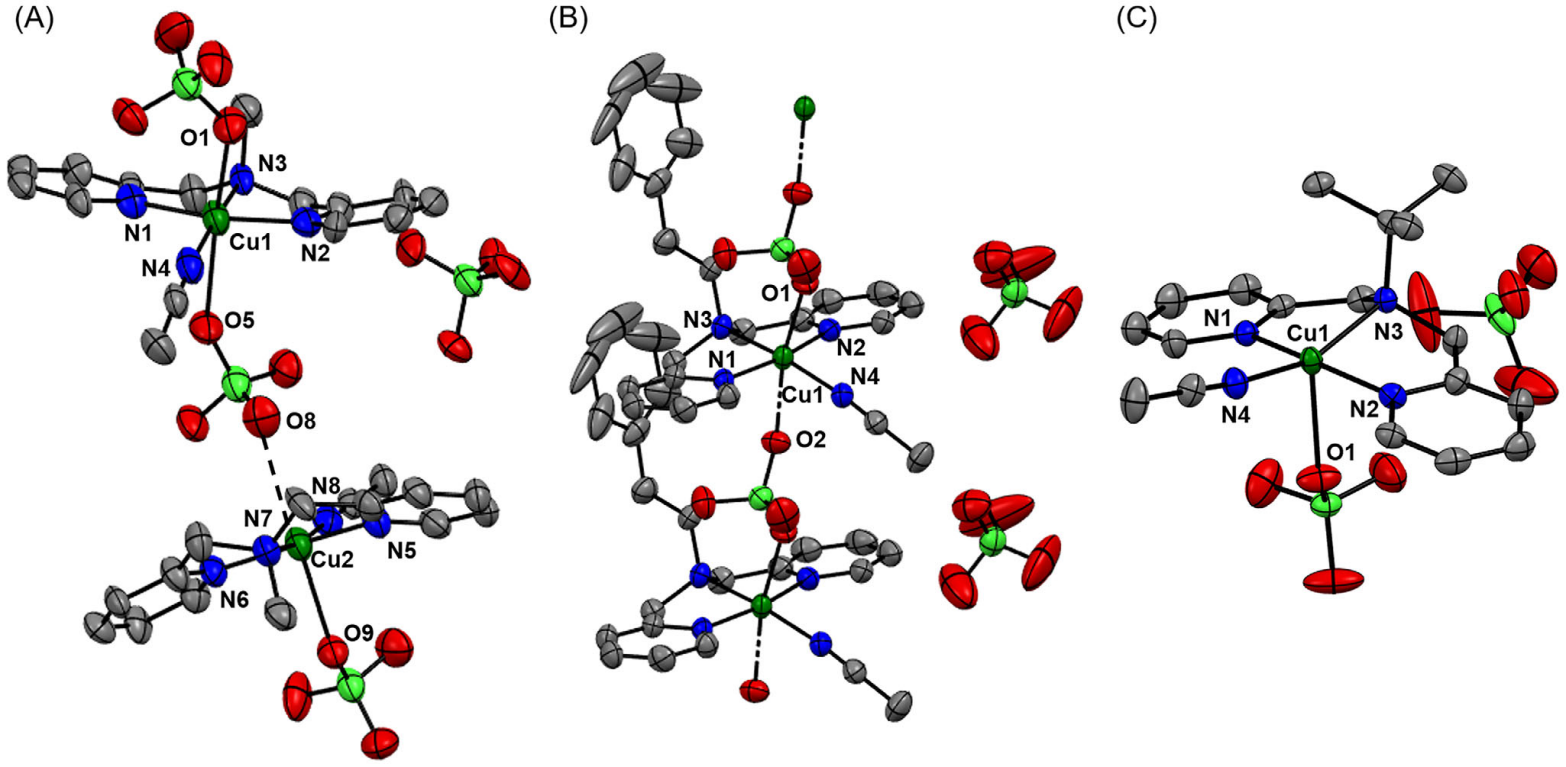
ORTEP plots of the molecular structures of **1^Me^** (A), **1^Phe^** (B), and **1*^t^* ^‐Bu^** (C). Carbon‐bound H‐atoms are omitted for clarity. Ellipsoids are drawn at 50% probability.

The N_3_‐tridentate ligands Rdpa (R = Me, Phe, *t*‐Bu) reacted with Cu(ClO_4_)_2_•6H_2_O to give [Cu(Rdpa)(MeCN)](ClO_4_)_2_ (**1^R^**) which were recrystallized from MeCN/Et_2_O (see Experimental Section). The crystal structures of **1^R^** were determined by x‐ray analysis. The molecular structures of these complexes are shown in Figure 1, and the crystallographic data and selected bond lengths and angles are listed in Tables S1–S4.

The asymmetric unit of **1^Me^** contains two complexes. In each complex, the Cu is coordinated by three N atoms from Medpa and one N atom from MeCN in the basal position, while an O atom of ClO_4_^−^ is coordinated in the apical position from the side where the Me group is located. Moreover, the ClO_4_^−^ between the two complexes binds to Cu1 and weakly bridges with Cu2, resulting in Cu1 and Cu2 adopting nonequivalent, axially elongated octahedral structures. The asymmetric unit of **1^Phe^** contains one complex. The Cu is coordinated by Phedpa and MeCN in the basal position in the same manner as **1^Me^**, and ClO_4_^−^ is apically coordinated from the side where the Phe group is located. The ClO_4_^−^ also bridges to the Cu of the other complex, forming a one‐dimensional chain structure. The Cu adopts an axially elongated octahedral structure. In the case of **1*^t^* ^‐Bu^**, although the Cu is coordinated by *t*‐Budpa and MeCN like **1^Me^** and **1^Phe^**, ClO_4_^−^ does not exist on the same face as the *t*‐Bu group but coordinates apically from the opposite face, adopting square pyramidal geometry. Thus, while **1^Me^** and **1^Phe^** form bridging structures, **1*^t^* ^‐Bu^** does not, clearly demonstrating that the steric effect of an alkyl group on the tertiary amine significantly influences the bridge formation. The average Cu─O bond length 2.483 Å in **1^Phe^** is shorter than that 2.602 Å in **1^Me^**, indicating that the Phe group is less sterically hindered to the ClO_4_^−^ bridge than the Me group. The bond lengths between Cu and tertiary amine (Cu─N_tert_) in **1^Me^**, **1^Phe^**, and **1*^t^* ^‐Bu^** are 2.004 (average), 2.048, and 2.038 (Å), respectively. The longest Cu–N_tert_ distance of **1^Phe^** may be due to distortion by one‐dimensional chain structure, but not the steric hindrance.

### 3.2 CyH Oxidation With H_2_O_2_ Catalyzed by **1^R^** (**1^Me^**, **1^Phe^**, **1*^t^* ^‐Bu^**)

**SCHEME 1:**
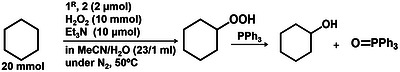
CyH oxidation with H_2_O_2_ catalyzed by **1^R^** or **2**.

The CyH oxidation with H_2_O_2_ catalyzed by **1^R^** was examined under reaction conditions shown in Scheme 1. The details of the reaction analyses are described in the Experimental Section. The main product in this reaction was cyclohexyl hydroperoxide (CyO_2_H), which was converted to cyclohexanol (CyOH) by the treatment with PPh_3_ for the GC analysis.

**FIGURE 2:**
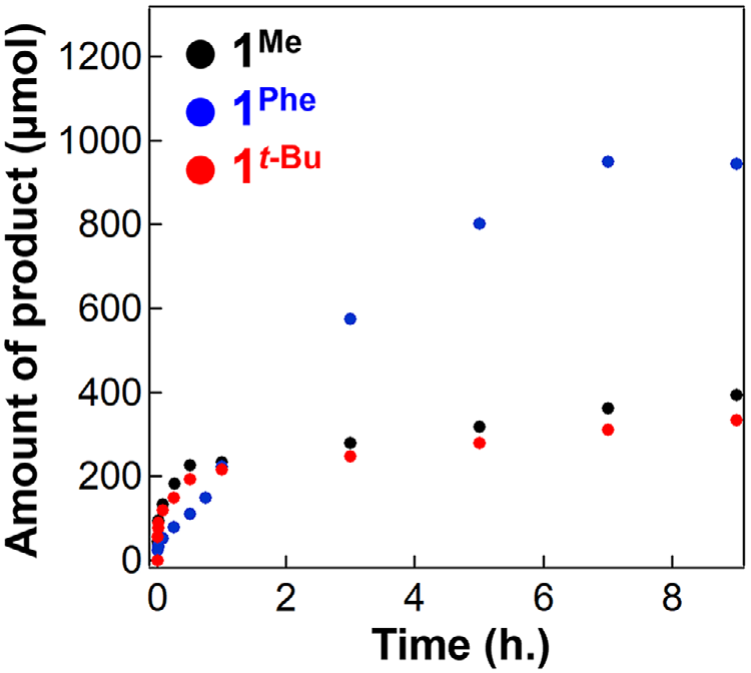
Time courses in the oxidation of CyH (20 mmol) with H_2_O_2_ (10 mmol) catalyzed by **1^R^** (2 mmol) in MeCN (23 mL) under N_2_ at 50°C.

Time courses of the product formation in the CyH oxidations catalyzed by **1^R^** are shown in Figure 2. Additionally, time courses in the initial reactions are shown in Figure S1, where the data of [Cu(tpa)(MeCN)](ClO_4_)_2_ (**2**), Cu(II) complex of tpa N_4_‐tetradentate ligand, are also shown to compare with **1^R^**. Turnover frequencies TOF (min^−^^1^) 92, 49, and 112 of **1^Me^**, **1^Phe^**, and **1*^t^* ^‐Bu^** are 21, 11, and 25 times higher, respectively, than 4.5 of **2**. As shown in Figure 2, however, the catalytic activity of **1^Me^** and **1*^t^* ^‐Bu^** rapidly ceased, but that of **1^Phe^** was rising for an extended period. The rapid decay of **1^Me^** and **1*^t^* ^‐Bu^** is caused by nonselective oxidations of the supporting ligands by HO• formed at a higher rate and in larger quantities than **2**. In fact, the CSI‐MS measured using aliquots taken from the reaction mixtures showed MS peaks at *m/z* = 303 and 262 (see Figure S2) corresponding to the oxidized ligands, where demethylation of Medpa and depyridylmethylation of *t*‐Budpa were detected.

**FIGURE 3:**
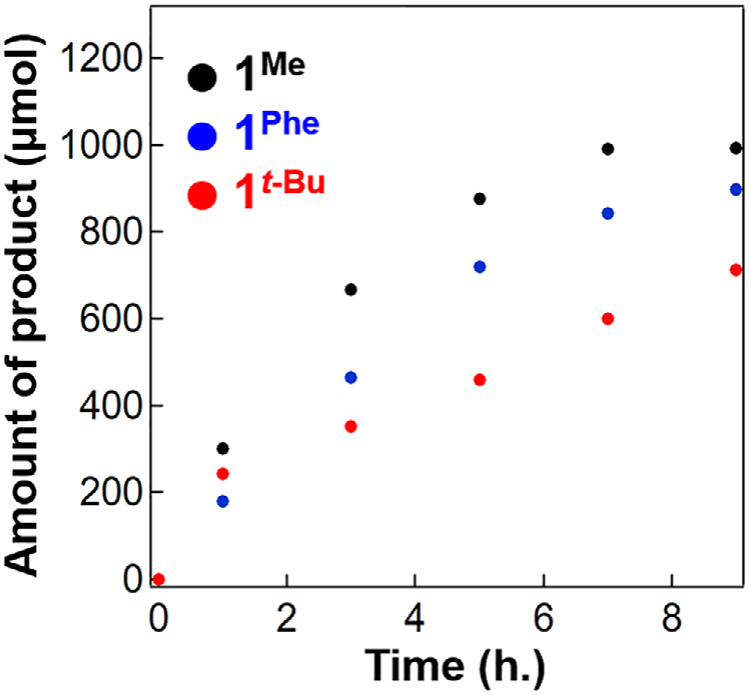
Time courses in the oxidation of CyH (20 mmol) with H_2_O_2_ (10 mmol) catalyzed by **1^R^** (2 mmol) in the presence of DMPO (120 µmol) in MeCN (23 mL) under N_2_ at 50°C.

To clarify the effect of HO• on the CyH oxidation, dimethylpyrrole‐*N*‐oxide (DMPO) [36] was used as a HO• trapping reagent in the reactions catalyzed by **1^Me^**, **1^Phe^**, and **1*^t^* ^‐Bu^**. The results are shown in Figure 3. For the reaction with **1^Me^** in the presence of DMPO, the initial rapid formation and saturation of products disappeared, and the products continued to increase over a prolonged period, significantly improving the TON. This indicated that DMPO traps HO• formed in the initial reaction, thereby suppressing the oxidative degradation of **1^Me^**. Moreover, the continued increase of products indicated that a complex‐based active species not trapped by DMPO is simultaneously formed and contributes to the CyH oxidation. In contrast, the increase of the CyH oxidation product in the presence of DMPO was smaller for **1*^t^* ^‐Bu^**. This indicated that, in the reaction by **1*^t^* ^‐Bu^**, the main reaction is a Fenton‐type reaction to form HO•. Unlike **1^Me^** and **1*^t^* ^‐Bu^**, **1^Phe^** exhibited high catalytic activity regardless of the absence or presence of DMPO, showing that the involvement of HO• by Fenton‐type reaction is small for **1^Phe^**, and the CyH oxidation primarily proceeded by complex‐based active species.

### 3.3 Tracking the Reactions of **1^Me^**, **1^Phe^**, **1*^t^* ^‐Bu^** with H_2_O_2_ Applying the Stopped‐Flow Method

**FIGURE 4:**
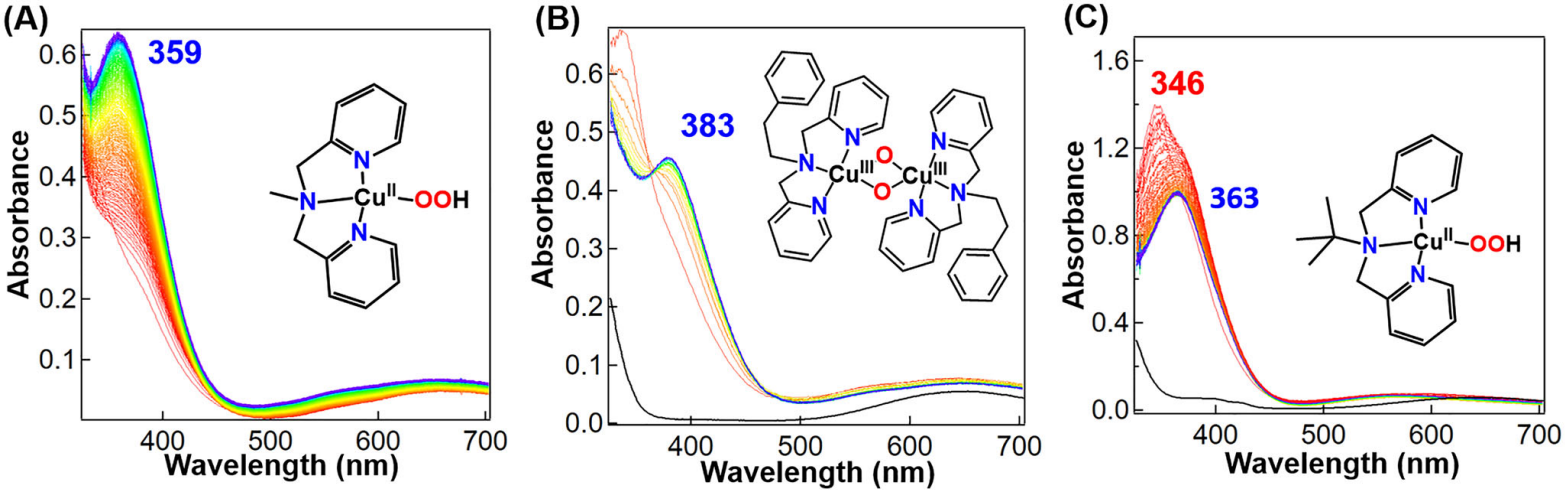
Spectral change from red to blue in the beginning of reaction of (A) **1^Me^** (3.5 ms to 3.5 s), (B) **1^Phe^** (2.5 ms to 0.25 s), and (C) **1*^t^* ^‐Bu^** (2.5 ms to 2.5 s) (0.5 mM) with H_2_O_2_ (2.5 mM) in the presence of Et_3_N (2.5 mM) in acetone at −80°C. The spectra were measured by the stopped‐flow technique. The chemical structure of the assigned intermediate is shown on each spectrum.

The reactivity of **1^R^** in the CyH oxidation with H_2_O_2_ differs depending on the R group of Rdpa. In the case of **1^Me^**, HO• and the complex‐based active species are formed concurrently. In cases of **1*^t^* ^‐Bu^** and **1^Phe^**, HO• and the complex‐based active species are the main active species, respectively. These should depend on the difference of H_2_O_2_‐activation by **1^Me^**, **1^Phe^**, and **1*^t^* ^‐Bu^**. To clarify these differences, the reactions of **1^R^** with H_2_O_2_ were monitored by the UV‐vis spectra on low‐temperature stopped‐flow methods to investigate the intermediates formed in the initial stage of the reactions. At −80°C in the presence of Et_3_N, **1^Me^**, **1^Phe^**, and **1*^t^* ^‐Bu^** reacted with H_2_O_2_ to give characteristic absorption bands at 359, 383, and 346 nm, respectively, as shown in Figure 4.

It was reported that [Cu(II)(*t*‐Budpa)O_2_H]^+^ and [Cu(III)_2_(Phedpa)_2_(μ‐O)_2_]^2^^+^ formed from **1*^t^* ^‐Bu^** with H_2_O_2_ and from a Cu(I) Phedpa complex with O_2_ (see Chart 2) show absorption bands at 350 and 385 nm, respectively [28, 30]. These bands are close to 346 and 383 nm observed in the stopped‐flow measurements for the reactions of **1*^t^* ^‐Bu^** and **1^Phe^** with H_2_O_2_, respectively (Figure 4B, C). This indicated that [Cu(II)(O_2_H)(*t*‐Budpa)]^+^ and [Cu(III)_2_(μ‐O)_2_(Phedpa)_2_]^2^^+^ are formed from **1*^t^* ^‐Bu^** and **1^Phe^** in the initial reactions with H_2_O_2_.

**FIGURE 5:**
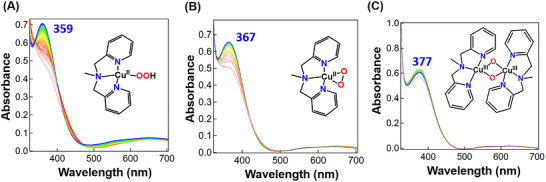
Spectral change from red to blue in the beginning of reaction of **1^Me^** (0.5 mM) with H_2_O_2_ (2.5 mM) in the presence of Et_3_N (2.5 mM) in acetone at (A) −80°C (3.5 ms to 3.5 s), (B) −55°C (3.5 ms to 0.35 s), and (C) −30°C (3.5 ms to 0.35 s). The spectra were measured by the stopped‐flow technique. The chemical structure of the assigned intermediate is shown on each spectrum.

**SCHEME 2:**
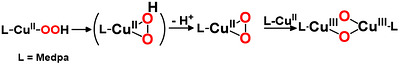
Formation of [Cu(III)_2_(μ‐O)_2_(Medpa)_2_]^2+^ in the reaction of **1^Me^** with H_2_O_2_.

However, since spectroscopic identification of intermediates formed from **1^Me^** with H_2_O_2_ has not been reported, the intermediates were examined by the low‐temperature stopped‐flow measurements at −80, −55, and −30°C (see Figure 5). The absorption band appeared at 359 nm in the reaction at −80°C and shifted to 367 nm and 377 nm when the temperature was raised to −55°C and −30°C, respectively. Itoh reported that in the reaction of [Cu(II)(Ph_2_edpea)]^2^^+^ with H_2_O_2_ (see Chart 2), [Cu(II)(Ph_2_edpea)O_2_H]^+^ (360 nm) is initially formed and deprotonated to give [Cu(II)(Ph_2_edpea)(μ‐η^1^:η^1^‐O_2_)] (366 nm), which reacts with another [Cu(II)(Ph_2_edpea)]^2^^+^ to form [Cu(II)_2_(Ph_2_edpea)_2_(μ‐η^2^:η^2^‐O_2_)]^2+^ (366 nm) [29]. The absorption bands at 359 and 365 nm observed at −80°C and −55°C in the reaction of **1^Me^** with H_2_O_2_ are similar to those of 360 and 366 nm of [Cu(II)(Ph_2_edpea)O_2_H]^+^ and [Cu(II)(Ph_2_edpea)(μ‐η^1^:η^1^‐O_2_)] and thus can be assigned to [Cu(II)(Medpa)O_2_H]^+^ and [Cu(II)(Medpa)(μ‐η^1^:η^1^‐O_2_)], respectively. However, the band at 377 nm observed at −30°C significantly differs from 366 nm of [Cu(II)_2_(Ph_2_edpea)_2_(μ‐η^2^:η^2^‐O_2_)]^2^^+^, rather close to that at 383 nm of [Cu(III)_2_(Phedpa)_2_(μ‐O)_2_]^2^^+^ [30]. These showed that in the reaction of **1^Me^** with H_2_O_2_, [Cu(II)O_2_H(Medpa)]^+^ is initially formed and converted to [Cu(III)_2_(μ‐O)_2_(Medpa)_2_]^2+^ via [Cu(II)(μ‐η^1^:η^1^‐O_2_)(Medpa)] as shown in Scheme 2.

**FIGURE 6:**
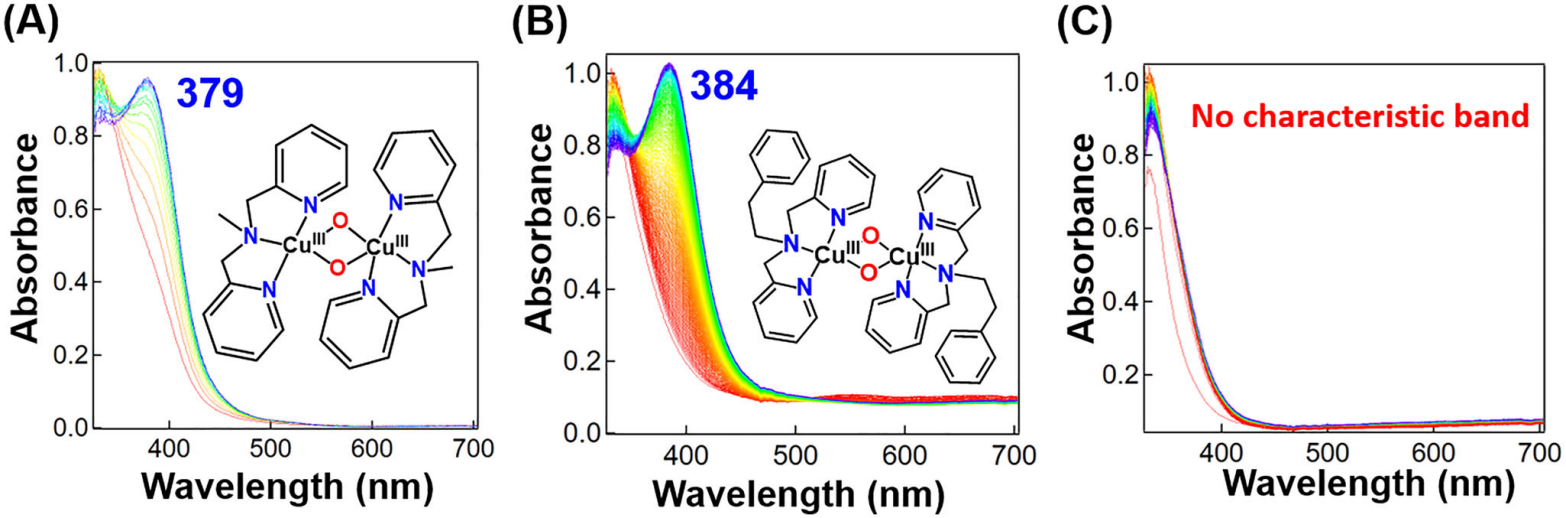
Spectral change from red to blue in the beginning of the reaction of Cu(I) complexes (0.5 mM) of Medpa (A) (3.5 ms to 15 ms), Phedpa (B) (2.5 ms to 2.0 s), and *t*‐Budpa (C) (2.5 ms to 2.5 s) with O_2_ in acetone at ‐80°C. The spectra were measured by the stopped‐flow technique. Assigned chemical species were shown on each spectrum.

Moreover, the reactions of Cu(I) complexes of Medpa, Phedpa, and *t*‐Budpa with O_2_ at −80°C were traced by the low‐temperature stopped‐flow method as shown in Figure 6. Itoh reported that upon reaction of the Cu(I) Phedpa complex with O_2_, [Cu(III)_2_(Phedpa)(μ‐O)_2_]^2+^ is formed (see Chart 2) and gives the band at 385 nm [30]. In this study, the same band appeared at 384 nm upon reaction of the Phedpa Cu(I) complex with O_2_ (Figure 6B) and at 383 nm upon reaction of **1^Phe^** with H_2_O_2_ (Figure 4B), clearly showing that [Cu(III)_2_(Phedpa)(μ‐O)_2_]^2+^ is formed by these reactions. In the same manner, the band at 379 nm observed upon reaction of the Medpa Cu(I) complex with O_2_ (Figure 6A) is assignable to [Cu(III)_2_(Medpa)(μ‐O)_2_]^2+^. This is almost the same as the band at 377 nm observed upon reaction of **1^Me^** with H_2_O_2_ at −30°C (Figure 5C). These results emphasized that [Cu(III)_2_(Medpa)(μ‐O)_2_]^2+^ and [Cu(III)_2_(Phedpa)(μ‐O)_2_]^2+^ are formed in the reactions of **1^Me^** and **1^Phe^** with H_2_O_2_, respectively. However, any characteristic absorption band was not observed upon reaction of the Cu(I) *t*‐Budpa complex with O_2_ (Figure 6C). This suggested that the formation of bis‐μ‐oxo Cu(III)_2_ with *t*‐Budpa is difficult due to the steric hindrance of the *t*‐Bu group.

As shown in the crystal structures, **1^Me^** and **1^Phe^** formed ClO_4_^−^ bridges, but **1*^t^* ^‐Bu^** did not due to the steric hindrance of the *t*‐Bu group. This indicated that the ease of bridging structure formation in **1^R^** is significantly influenced by the steric hindrance of R group. This is consistent with the fact that **1^Me^** and **1^Phe^** form the bis‐μ‐oxo Cu(III)_2_, but **1*^t^* ^‐Bu^** only forms the hydroperoxo Cu(II). Moreover, since the Cu─O bond in the ClO_4_^−^ bridge is shorter in **1^Phe^** than in **1^Me^**, the Phe group is more favorable to form the bridging structure than the Me group. This is the reason why the bis‐μ‐oxo Cu(III)_2_ is formed from **1^Phe^** as the sole detectable intermediate upon reaction with H_2_O_2_, but both the hydroperoxo Cu(II) and the bis‐μ‐oxo Cu(III)_2_ from **1^Me^**.

### 3.4 H_2_O_2_ Activation Pathways by **1^R^**

**FIGURE 7:**
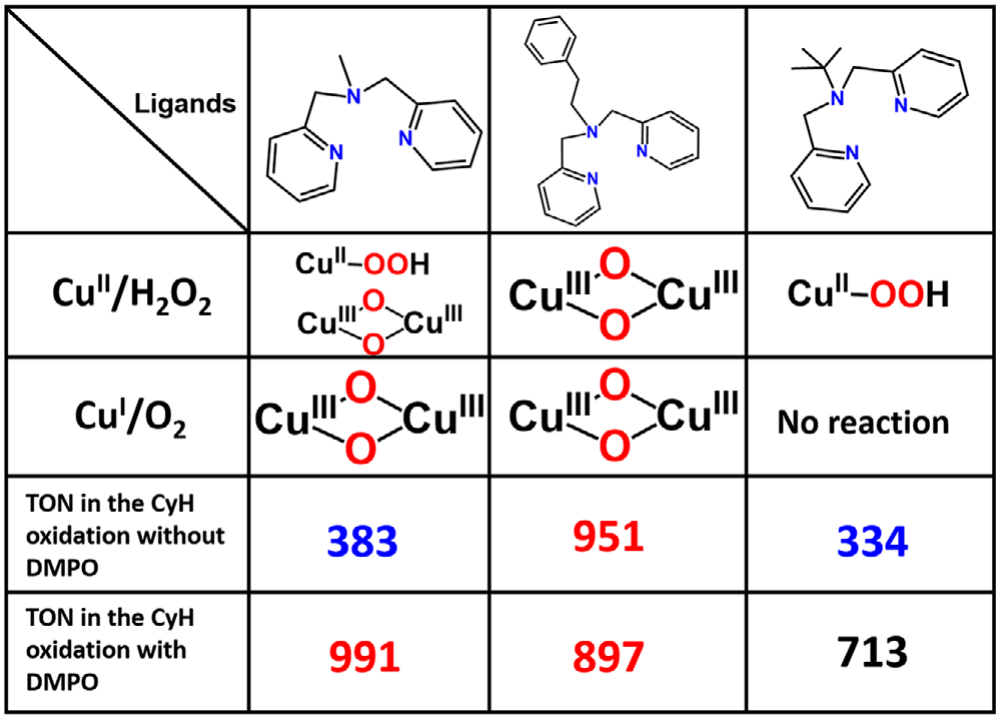
Intermediates formed in the reactions of **1^R^** with H_2_O_2_ or of the Cu(I) Rdpa complexes with O_2_ and TONs of **1^R^** in the CyH oxidations in the absence and presence of DMPO.

Figure 7 shows intermediates formed in the reactions of **1^R^** with H_2_O_2_ or of the Cu(I) Rdpa complexes with O_2_ and TONs of **1^R^** in the CyH oxidations with H_2_O_2_ in the absence and presence of DMPO. In the case of **1^Me^**, both hydroperoxo Cu(II) and bis‐μ‐oxo Cu(III)_2_ intermediates are formed, and the catalytic activity in the CyH oxidation is improved by DMPO trapping HO•. In the case of **1^Phe^**, the bis‐μ‐oxo Cu(III)_2_ intermediate is solely detected, and a large TON is attained in the CyH oxidation, whether DMPO exists or not. In the case of **1*^t^* ^‐Bu^**, the hydroperoxo Cu(II) intermediate is solely detected, and TON is not so improved by DMPO. These results suggested that hydroperoxo Cu(II) and bis‐μ‐oxo Cu(III)_2_ intermediates are converted to HO• and complex‐based active species, respectively.

**SCHEME 3:**
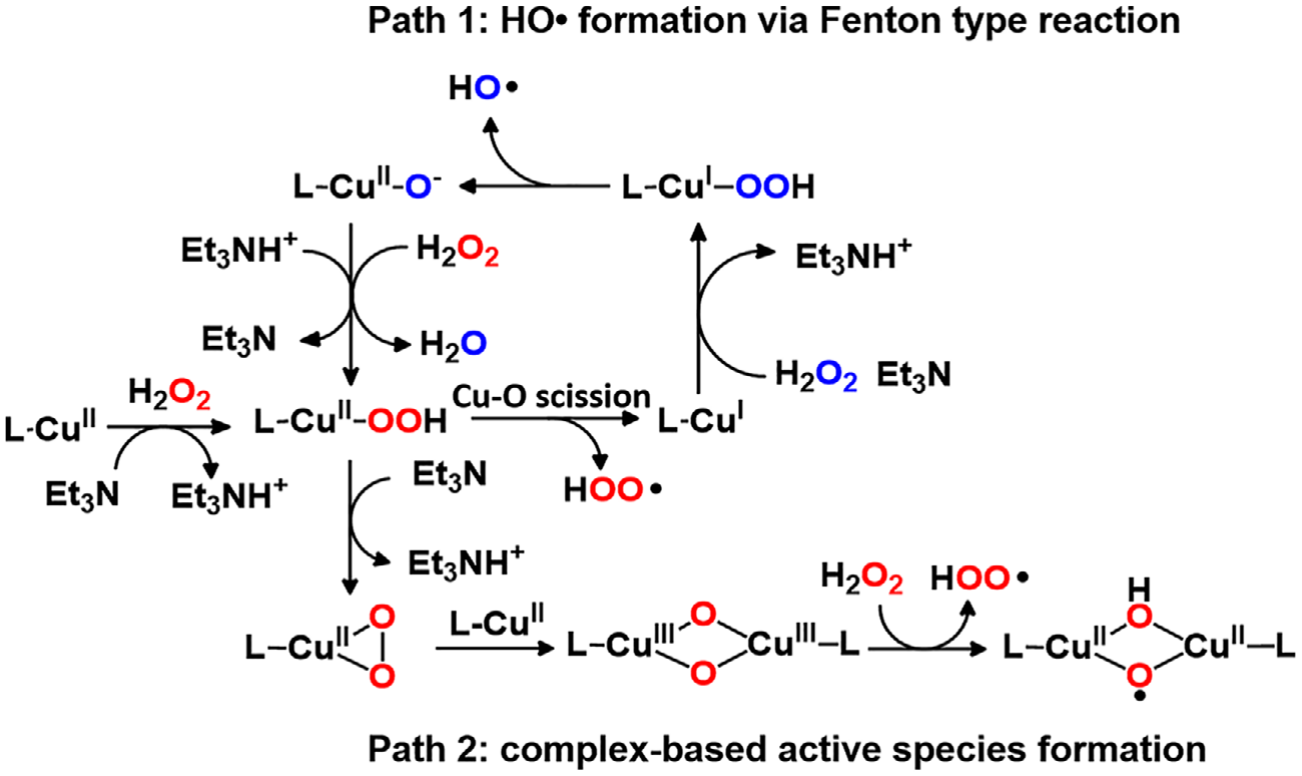
Proposed mechanism for the Paths 1 and 2 in the H_2_O_2_ activation by **1^R^**.

Therefore, the hydroperoxo Cu(II) intermediates formed from **1^Me^** and **1*^t^* ^‐Bu^** cause Fenton‐type reactions to give HO•, which nonselectively oxidizes supporting ligands. This reaction pathway to form HO• via the hydroperoxo Cu(II) intermediate is shown as Path 1. Meanwhile, it has been reported that the bis‐μ‐oxo Cu(III)_2_ intermediates intramolecularly oxidize the supporting ligand C─H bonds [38]. In this study, the oxidation of the Phenethyl group of **1^Phe^** was shown by the CSI‐MS measurement on the reaction of **1^Phe^** with H_2_O_2_ (see Figure S2). However, the bis‐μ‐oxo Cu(III)_2_ complex cannot be a complex‐based active species in the CyH oxidation because it does not have the ability to oxidize a strong C─H bond having a large BDE intermolecularly [39, 40]. Therefore, it is proposed that a μ–O• bridged Cu(II)_2_ is formed as a relevant complex‐based active species via H‐atom transfer from excess amounts of H_2_O_2_ to the bis‐μ‐oxo Cu(III)_2_ intermediate. Itoh reported the involvement of a μ–O• bridged Cu(III)_2_ complex in the substrate oxidation based on the kinetic evidence without spectroscopic identification [41]. Moreover, the μ‐O• bridged di‐ and tricopper species were proposed based on the DFT studies [20, 22, 23, 24]. The reaction pathway that the bis‐μ‐oxo Cu(III)_2_ intermediate is converted to a relevant μ–O• bridged Cu(II)_2_ active species is shown as Path 2. The Paths 1 and 2 are shown in Scheme 3.

### 3.5 Control of H_2_O_2_ Activation Pathway by **1^Me^**

**FIGURE 8:**
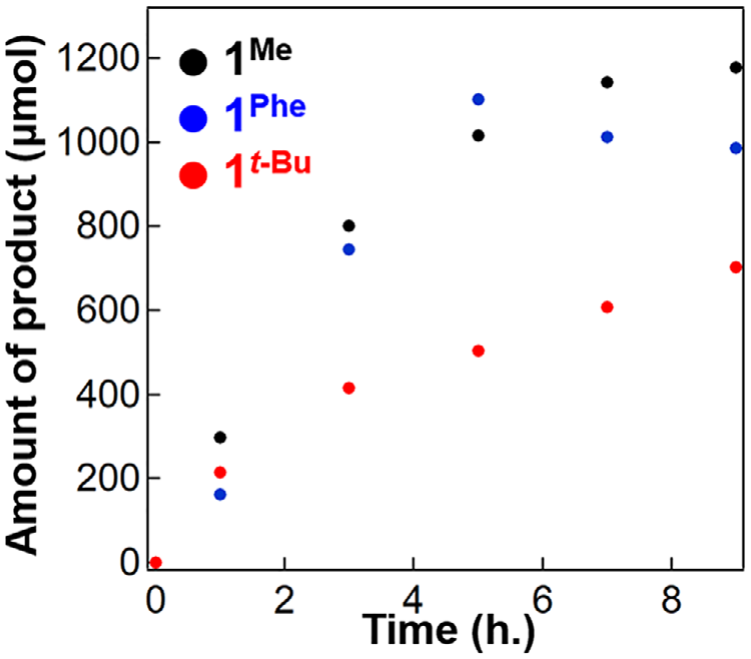
Time courses in the CyH oxidation with H_2_O_2_ (10 mmol) catalyzed by **1^R^** (2 µmol) in MeCN at 50°C. H_2_O_2_ was added stepwise (H_2_O_2_ 1 mmol × 10, every 5 min).

The hydroperoxo Cu(II) intermediate is formed at a 1:1 ratio of **1^Me^** to H_2_O_2_, while the bis‐μ‐oxo Cu(III)_2_ intermediate is formed at a 2:1 ratio. Thus, it is considered that the bis‐μ‐oxo Cu(III)_2_ intermediate is favorably formed under lower H_2_O_2_ concentration. So, the CyH oxidation by **1^Me^** was carried out under conditions where the steady‐state H_2_O_2_ concentration is reduced by adding 1 mmol of H_2_O_2_ 10 times at 5 min intervals, instead of adding 10 mmol of H_2_O_2_ all at once. The results are shown in Figure 8. Under low H_2_O_2_ concentration, **1^Me^** was not deactivated rapidly, and the TON largely increased, indicating that reducing the steady‐state H_2_O_2_ concentration shifts the main pathway from Path 1 to Path 2 and suppresses the oxidative decomposition of the catalyst by HO•. Thus, the formation of complex‐based active species by **1^Me^** depends on H_2_O_2_ concentration. In contrast, no significant increase in the oxidation products was observed for **1^Phe^** and **1*^t^* ^‐Bu^** when the steady‐state H_2_O_2_ concentration was lowered. This supports that each main reaction pathway of **1^Phe^** and **1*^t^* ^‐Bu^** in the CyH oxidation is Path 2 and Path 1, respectively.

### 3.6 Effect of Redox Potential of **1^R^** on the H_2_O_2_ Activation Pathway

To investigate the effect of the redox potential on the catalytic activity of **1^R^**, the cyclic voltammograms of **1^R^** and **2** were measured (see Figures S3 and S4, and Table S5). The Cu(I)/Cu(II) redox potentials of **1^Me^**, **1^Phe^**, and **1*^t^* ^‐Bu^** are −0.277, −0.214, and −0.098 V, respectively, showing a positive shift in potential with increasing bulkiness of the R group. These values are much more positive than the Cu(I)/Cu(II) redox potential of −0.413 V for **2** [42]. Thus, the N_3_‐tridentate ligand Rdpa stabilizes the Cu(I) state more than the N_4_‐tetrahedral ligand tpa. This is the reason why large amounts of HO• are formed at the beginning of the H_2_O_2_ activation by **1^Me^** and **1*^t^* ^‐Bu^**, resulting in a sharp decrease of the catalytic activity by nonselective ligand oxidations. This was not observed in the reaction by **1^Phe^** despite the slightly more positive Cu(I)/Cu(II) redox potential of **1^Phe^** compared with that of **1^Me^**. This is probably because **1^Phe^** rapidly forms the bis‐μ‐oxo Cu(III)_2_ intermediate to promote Path 2. Moreover, even if a Cu(I) complex is formed from **1^Phe^** via Path 1, the Cu(I) is coordinated by the phenyl group as reported by Itoh [43], which potentially hinders the reaction of the Cu(I) complex with H_2_O_2_ required for the Fenton‐type reaction.

## 4 Conclusions

The catalytic activities of **1^R^**, Cu(II) complexes with N_3_‐tridentate ligands Rdpa (R = Me, Phe, and *t*‐Bu), in the CyH oxidation with H_2_O_2_ were examined by detailed reaction analyses. The CyH oxidation by **1^R^** proceeded via two reaction pathways, a Fenton‐type reaction forming HO• as the active species (Path 1) and a reaction of a complex‐based active species (Path 2). Reaction intermediates formed upon reaction of **1^R^** with H_2_O_2_ were detected by low‐temperature stopped‐flow techniques. Both hydroperoxo Cu(II) and bis‐μ‐oxo Cu(III)_2_ were formed from **1^Me^** as key intermediates. Meanwhile, hydroperoxo Cu(II) was formed from **1*^t^* ^‐Bu^**, and bis‐μ‐oxo Cu(III)_2_ was from **1^Phe^** as the sole detectable intermediate. In Path 1, HO• is formed via the hydroperoxo Cu(II) intermediate, resulting in rapid degradation of the catalyst by nonselective ligand oxidation by HO• to decrease the TON. In Path 2, a relevant complex‐based μ–O• bridged Cu(II)_2_ active species is formed via the bis‐μ‐oxo Cu(III)_2_ and elongates the catalyst life to increase the TON. Thus, since **1^Me^** catalyzes both Paths 1 and 2 via the hydroperoxo Cu(II) and the bis‐μ‐oxo Cu(III)_2_ intermediates, the catalytic performance of **1^Me^** is largely enhanced by decreasing HO• using DMPO HO• trapping reagent and by promoting the formation of bis‐μ‐oxo Cu(III)_2_ intermediate under low H_2_O_2_ concentration. **1*^t^* ^‐Bu^** only forms the hydroperoxo Cu(II) intermediate and catalyzes Path 1, resulting in relatively low TON. Meanwhile, **1^Phe^** rapidly forms the bis‐μ‐oxo Cu(III)_2_ intermediate and mainly catalyzes Path 2, giving a large TON. These show that the bis‐μ‐oxo Cu(III)_2_ is a key intermediate to achieve large TON in the CyH oxidation. Therefore, it is concluded that the ease of dinuclear structure formation in the Cu complexes with N_3_‐tridentate ligands is a key feature to enhance the catalytic activity in the alkane oxidation with H_2_O_2_. The results are helpful for the future design of ligands for copper complexes that could be applied in alkane oxidation reactions.

## Author Contributions

The manuscript was written through the contributions of all authors. All authors have given approval to the final version of the manuscript.

## Conflicts of Interest

The authors declare no conflicts of interest.

## Supporting information



The Supporting Information is available free of charge on the ACS Publications website: Tables S1‐S5, Figures S1–S4, and x‐ray crystallographic data (CIF).

## Data Availability

The data that support the findings of this study are available from the corresponding author upon reasonable request.
