# Supplementary material for: H2O2 Activation and Alkane Oxidation by Copper Complexes With R‐dpa N3‐Tridentate Ligands: The Complex‐Based Dicopper Active Species as a Key Feature in the Efficient Alkane Oxidation
Source: Chemistry. 2026 Apr 16;32(24):e70985. doi: 10.1002/chem.70985 (PMC13290420; doi:10.1002/chem.70985)
Supplement: Supplementary file 1 — The Supporting Information is available free of charge on the ACS Publications website: Tables S1‐S5, Figures S1–S4, and x‐ray crystallographic data (CIF). [file CHEM-32-e70985-s001.pdf]

**Supporting Information**

# $H_2O_2$ Activation and Alkane Oxidation by Copper Complexes with R-dpa $N_3$ Tridentate Ligands: The Complex-Based Dicopper Active Species as a Key Feature in the Efficient Alkane Oxidation

Kyosuke Fujikawa[†], Momoe Kawahashi[†], Alexander Granichny[§], Siegfried Schindler[§], Hiroaki Kitagishi[†], and Masahito Kodera[†*]

† Department of Molecular Chemistry and Biochemistry, Doshisha University, 1-3 Tatara Miyakodani, Kyotanabe, Kyoto 610-0321, Japan

§ Institute of Inorganic and Analytical Chemistry, Justus Liebig University Gessen, Gessen, Hessen 35329, Germany

*E-mail: mkodera@mail.doshisha.ac.jp

**Table S1.** Crystallographic data of $\mathbf{1}^{\mathbf{R}}$.

| | $\mathbf{1}^{\mathbf{Me}}$ | $\mathbf{1}^{\mathbf{Phe}}$ | $\mathbf{1}^{\boldsymbol{t}\mathbf{\text{-}Bu}}$ |
|---|---|---|---|
| Empirical formula | $C_{15}H_{18}Cl_2CuN_4O_8$ | $C_{22}H_{24}Cl_2CuN_4O_8$ | $C_{18}H_{24}Cl_2CuN_4O_8$ |
| Formula weight | 516.78 | 606.90 | 558.86 |
| Temperature (K) | 103 | 103 | 103 |
| Wavelength | CuKα (1.54187Å) | CuKα (1.54187Å) | CuKα (1.54187Å) |
| Crystal system | triclinic | monoclinic | monoclinic |
| Crystal size ($mm^3$) | 0.19 × 0.04 × 0.03 | 0.069 × 0.244 × 0.023 | 0.339 × 0.110 × 0.066 |
| Space group | P-1 | $P2_1$ / c | $P2_1$ / c |
| A (Å) | 12.1261(5) | 7.1904 (3) | 8.3574 (3) |
| B (Å) | 13.5046(6) | 13.9603 (5) | 14.1771 (4) |
| C (Å) | 13.6382(6) | 25.7397 (9) | 19.8256 (6) |
| α (deg) | 86.830(6) | 90 | 90 |
| β (deg) | 65.601(5) | 97.910 (7) | 100.106 (7) |
| γ (deg) | 79.328(6) | 90 | 90 |
| Volume ($Å^3$) | 1998.18(18) | 2559.17 (17) | 1.605 |
| Z value | 4 | 4 | 4 |
| Density (calculated) | 1.718 g/cm3 | 1.570 g/cm3 | 1.483 g/cm3 |
| Absorption coefficient | 4.491 mm 1 | 3.605 mm 1 | 3.928 mm 1 |
| F(000) | 1050.4 | 1242.9 | 1146.6 |
| R1[a)], wR2[b)] | 0.0862, 0.2223 | 0.0622, 0.1915 | 0.0441, 0.1244 |
| GOF index | 1.000 | 1.014 | 1.047 |

a) $R1 = \Sigma||Fo| - |Fc|/ \Sigma|Fo|$, b) $wR2 = [\Sigma(w(Fo^2 - Fc^2)^2)/ \Sigma w(Fo^2)^2]^{1/2}$

**Table S2.** Selected bond distances (Å) and angles (deg) for $\mathbf{1}^{\mathbf{Me}}$.

| Bond distances (Å) | | | |
|---|---|---|---|
| Cu1–N1 | 1.985 | Cu2–N5 | 1.977 |
| Cu1–N2 | 1.970 | Cu2–N6 | 1.985 |
| Cu1–N3 | 1.999 | Cu2–N7 | 2.009 |
| Cu1–N4 | 1.983 | Cu2–N8 | 1.953 |
| Cu1–O1 | 2.530 | Cu2–O8 | 2.851 |
| Cu1–O5 | 2.582 | Cu2–O9 | 2.445 |
| **Bond angles (deg)** | | | |
| N1–Cu1–N2 | 165.64 | N5–Cu2–N6 | 165.40 |

| | | | |
|---|---|---|---|
| N1–Cu1–N3 | 82.60 | N5–Cu2–N7 | 82.30 |
| N1–Cu1–N4 | 96.95 | N5–Cu2–N8 | 95.46 |
| N2–Cu1–N3 | 83.21 | N6–Cu2–N7 | 83.18 |
| N2–Cu1–N4 | 97.40 | N6–Cu2–N8 | 99.10 |
| N3–Cu1–N4 | 173.47 | N7–Cu2–N8 | 169.43 |
| O1–Cu1–N1 | 100.52 | O8–Cu2–N5 | 88.88 |
| O1–Cu1–N2 | 79.70 | O8–Cu2–N6 | 91.83 |
| O1–Cu1–N3 | 99.42 | O8–Cu2–N7 | 86.72 |
| O1–Cu1–N4 | 87.07 | O8–Cu2–N8 | 82.91 |
| O5–Cu1–N1 | 86.91 | O9–Cu2–N5 | 92.00 |
| O5–Cu1–N2 | 96.04 | O9–Cu2–N6 | 88.40 |
| O5–Cu1–N3 | 93.22 | O9–Cu2–N7 | 97.61 |
| O5–Cu1–N4 | 80.26 | O9–Cu2–N8 | 92.78 |
| O1–Cu1–O5 | 166.03 | O8–Cu2–O9 | 175.67 |

**Table S3.** Selected bond distances (Å) and angles (deg) for **1**$^{\mathbf{Phe}}$.

| Bond distances (Å) | | | |
|---|---|---|---|
| Cu–N1 | 1.964 | Cu–N4 | 1.993 |
| Cu–N2 | 1.974 | Cu–O1 | 2.444 |
| Cu–N3 | 2.048 | Cu–O2 | 2.523 |
| Bond angles (deg) | | | |
| N1–Cu–N2 | 167.11 | O1–Cu–N3 | 103.35 |
| N1–Cu–N3 | 83.82 | O1–Cu–N4 | 84.14 |
| N1–Cu–N4 | 97.99 | O2–Cu–N1 | 92.14 |
| N2–Cu–N3 | 83.47 | O2–Cu–N2 | 85.05 |
| N2–Cu–N4 | 95.33 | O2–Cu–N3 | 86.71 |
| N3–Cu–N4 | 172.46 | O2–Cu–N4 | 85.77 |
| O1–Cu–N1 | 91.28 | O1–Cu–O2 | 169.67 |
| O1–Cu–N2 | 93.68 | | |

**Table S4.** Selected bond distances (Å) and angles (deg) for **1**$^{\boldsymbol{t}\text{-}\mathbf{Bu}}$.

| Bond distances (Å) | | | |
|---|---|---|---|
| Cu1–N1 | 1.957 | Cu1–N4 | 1.988 |
| Cu1–N2 | 1.958 | Cu1–O1 | 2.402 |
| Cu1–N3 | 2.038 | | |

| Bond angles (deg) | | | |
|---|---|---|---|
| N1–Cu1–N2 | 164.53 | N3–Cu1–N4 | 157.29 |
| N1–Cu1–N3 | 85.22 | N1–Cu1–O1 | 87.89 |
| N1–Cu1–N4 | 96.77 | N2–Cu1–O1 | 82.73 |
| N2–Cu1–N3 | 85.42 | N3–Cu1–O1 | 105.30 |
| N2–Cu1–N4 | 96.62 | N4–Cu1–O1 | 97.39 |
| $\tau$ values | | | |
| $\tau$Cu = 0.121 | | | |

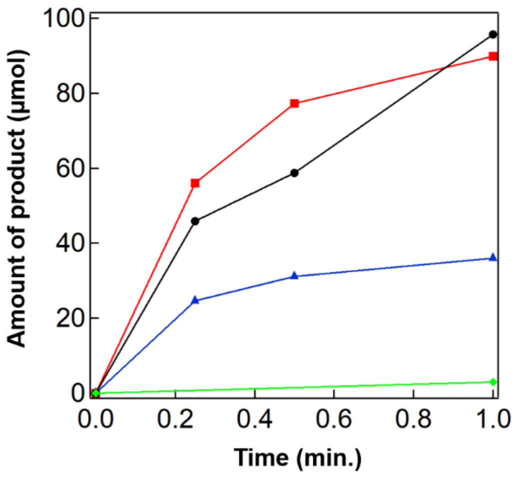


**Figure S1**. Time courses for CyH oxidation by **1**$^{Me}$ (black), **1**$^{t\text{-}Bu}$ (red), **1**$^{Phe}$ (blue) and **2** (green) in the early stage of the reaction. Reaction conditions were the same with Figure 2.

(a)

| m/z | |
|---|---|
| 276.07 | $[Cu^{I} + Medpa]^{+}$ |
| 303.22 | $[Cu^{I} + D + MeCN]^{+}$ |

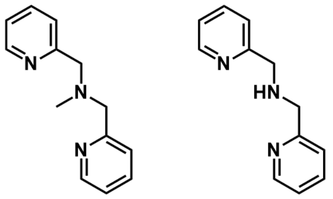

Medpa = 213.13 D = 199.11

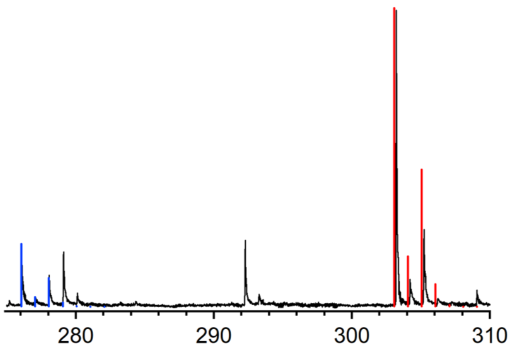


(b)

| m/z | |
|---|---|
| 366.10 | $[Cu^{I} + Phedpa]^{+}$ |
| 380.08 | $[Cu^{I} + K]^{+}$ |
| 394.07 | $[Cu^{I} + DK]^{+}$ |

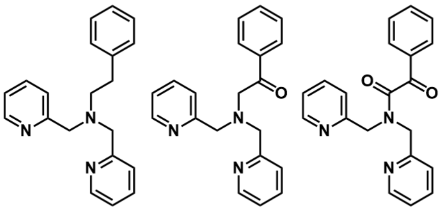

Phedpa = 303.17 K = 317.15 DK = 331.13

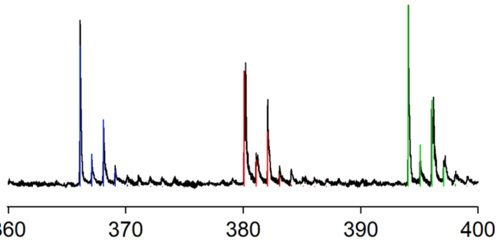


(c)

| m/z | |
|---|---|
| 266.07 | $[Cu^{I} + D + MeCN]^{+}$ |
| 318.11 | $[Cu^{I} + t\text{-}Budpa]^{+}$ |

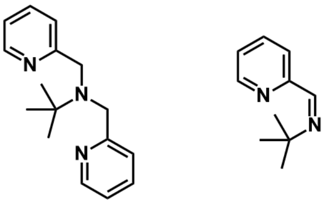

t-Budpa = 255.17 D = 162.12

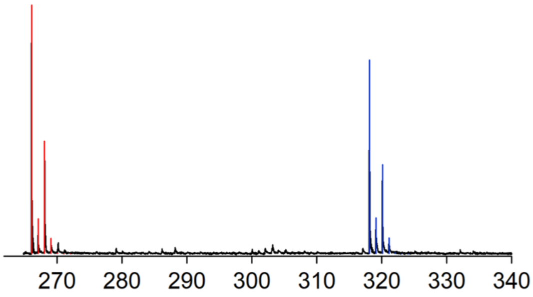


**Figure S2**. ESI-MS measurement for reaction solution of CyH oxidation with $H_2O_2$ catalyzed by $\mathbf{1}^{\mathbf{Me}}$ (a), $\mathbf{1}^{\mathbf{Phe}}$ (b) and $\mathbf{1}^{\boldsymbol{t}\text{-}\mathbf{Bu}}$ (c).

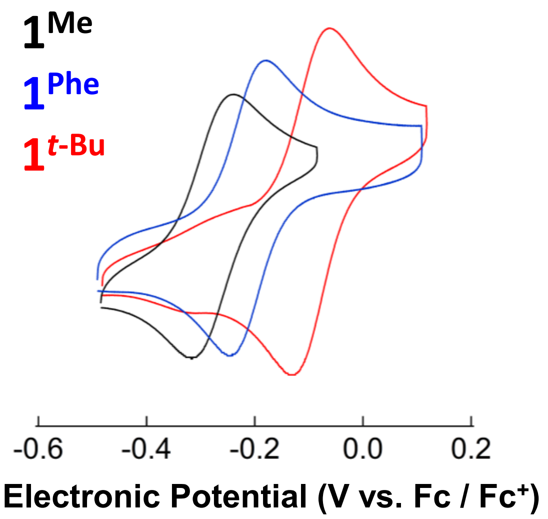


**Figure S3**. Cyclic Voltammogram of **1**$^{R}$ (0.1 mM) in MeCN TBAP (0.1 M) at room temperature. 3 mm GC working electrode (0.07 cm$^2$), Pt wire counter electrode, Ag/$AgNO_3$ reference electrode, Scan rate: 25 mV s$^{-1}$, Sensitivity: 1.0 mA / V, Segment 2.

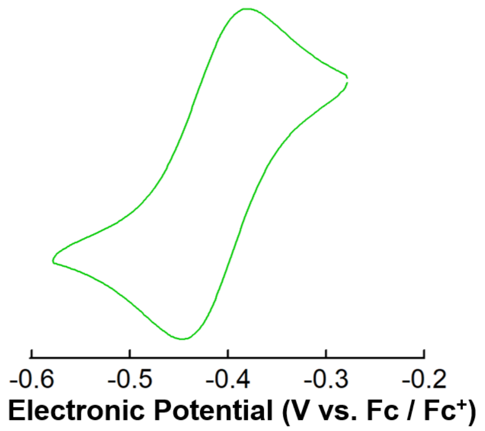


**Figure S4**. Cyclic Voltammogram of **2** (0.5 mM) in MeCN TBAP (0.1 M) at room temperature. 3 mm GC working electrode (0.07 cm$^2$), Pt wire counter electrode, Ag/$AgNO_3$ reference electrode, Scan rate: 25 mV s$^{-1}$, Sensitivity: 1.0 mA / V, Segment 2.

**Table S5**. Redox poyentials (V vs. Fc / Fc$^+$) of **1**$^{R}$ and **2**.

| | **1**$^{Me}$ | **1**$^{Phe}$ | **1**$^{t\text{-}Bu}$ | **2** |
|---|---|---|---|---|
| $E_{1/2}$ (V) | -0.277 | -0.215 | -0.098 | -0.413 |
